# Clinical and Image Characteristics of IgG4-Related Sclerosing Cholecystitis

**DOI:** 10.3390/diagnostics11081358

**Published:** 2021-07-28

**Authors:** Masaki Kuwatani, Naoya Sakamoto

**Affiliations:** Department of Gastroenterology and Hepatology, Hokkaido University Hospital, North 14, West 5, Kita-ku, Sapporo 060-8648, Japan; sakamoto@med.hokudai.ac.jp

**Keywords:** IgG4, sclerosing, cholecystitis, gallbladder, ultrasonography

## Abstract

Since autoimmune pancreatitis (AIP) was established as a new disease entity, sclerosing change with abundant immunoglobulin-4 (IgG4)-positive plasma cells, storiform fibrosis, and obliterative phlebitis are main pathological features in IgG4-related diseases. Regarding IgG4-related sclerosing cholecystitis (IgG4-CC), which is occasionally associated with AIP cases and is rarely isolated, there are no diagnostic criteria and insufficient perceptions of the image findings. Although there have been some reports on IgG4-CC, differentiation between IgG4-CC and gallbladder cancer is very difficult in some cases with a localized lesion. In this review, we especially focused on image findings of IgG4-CC and summarized its image features for diagnostic assistance. The ultrasonography and CT findings of IgG4-CC could be classified into diffuse and localized types. Based on these findings, the presence of wall thickening with an intact or smooth mucosal layer, followed by a homogenously thickened outer layer, would be a helpful morphological finding to distinguish IgG4-CC from gallbladder cancer.

## 1. Introduction

Since autoimmune pancreatitis (AIP) was established as a new entity of immune-related diseases, namely, immunoglobulin-4 (IgG4)-related sclerosing pancreatitis in 2002, there have been many reports and knowledge regarding AIP and systemic IgG4-related disease accumulated in Japan and around the world. Although the fundamental cause and detailed mechanism of IgG4-related disease, except for some candidates of antigens [1,2] and the involvement of B-cells [3,4], have not been clarified, steroid (glucocorticoid) administration has been a gold standard of the therapies, and sclerosing change with abundant IgG4-positive plasma cells (lymphoplasmacytic cells), storiform fibrosis, and obliterative phlebitis are the main pathological features [5,6]. For AIP, swelling of the pancreas with a capsule-like rim and long or multiple strictures of the main pancreatic duct without marked upstream dilatation are major image features [5]. To date, the diagnostic criteria of IgG4-related sclerosing cholangitis have been published, and have indicated that image features such as the specific biliary stricture patterns (type 1–4) and circular and symmetrical thickening of the bile duct wall with a smooth inner margin are characteristic findings [7]. Meanwhile, there have been some atypical cases with IgG4-related diseases that were difficult to differentiate from site-specific malignant diseases such as pancreatic cancer, bile duct cancer, lung cancer, submandibular adenocarcinoma, lymphoma, and gallbladder cancer [8]. There have been also some cases with both a malignant tumor and IgG4-positive cells/elevated serum IgG4 levels. Therefore, we need to pay close attention to the diagnosis of IgG4-related disease, to know its various image patterns, and to obtain pathological evidence before the decision regarding a treatment strategy, whenever possible.

Regarding IgG4-related sclerosing cholecystitis (IgG4-CC), which is associated with 25–32% of AIP [9] and is rarely isolated, there have been no diagnostic criteria and insufficient perceptions of the image findings. Although there have been some reports on IgG4-CC, differentiation between IgG4-CC and gallbladder cancer can be very difficult in some cases with a localized lesion.

Among the pathological features of IgG4-related diseases, dense lymphocyte and plasma cell infiltration, a ratio of IgG4-positive plasma cells/IgG-positive cells > 40% and the number of IgG4-positive plasma cells > 10/high powered field, and storiform fibrosis/obliterative phlebitis are common.

## 2. Diagnosis and Pathological Features of IgG4-Related Sclerosing Cholecystitis

There have not been the definite and specific criteria of IgG4-CC so far. Therefore, we need to follow the 2020 revised comprehensive diagnostic criteria for IgG4-related disease [10], as follows: (1) clinical and radiological features (one or more of the organs characteristic of IgG4-related disease), (2) serological diagnosis (serum IgG4 levels greater than 135 mg/dL), and (3) pathological diagnosis (positivity for two of the following three criteria: (1) dense lymphocyte and plasma cell infiltration with fibrosis, (2) ratio of IgG4-positive plasma cells/IgG-positive cells greater than 40% and the number of IgG4-positive plasma cells greater than 10 per high powered field, and (3) typical tissue fibrosis/particularly storiform fibrosis/obliterative phlebitis). For the definite diagnosis of IgG4-CC, all three of the abovementioned criteria have to be fulfilled. In addition, IgG4-CC can also be diagnosed by referring to the diagnostic criteria of AIP [5] or IgG4-related sclerosing cholangitis [7], mainly after resection of the gallbladder, through confirmation of a combination of the following: (1) characteristic image finding of the gallbladder, with a diffuse wall thickening/a nodule or mass with a smooth mucosal layer as described below, (2) elevated serum IgG4 level, (3) pathological findings along with the 2020 criteria, (4) other organ involvement, and (5) steroid treatment effect. Whichever criteria [5,7,10] are followed, it is still often difficult to fulfill the pathological criteria—to obtain sufficient and high-quality material without surgery.

According to the report regarding the pathology of IgG4-CC by Kamisawa et al. [11], there are two major patterns of lymphoplasmacytic infiltration, namely transmural lymphoplasmacytic infiltration with fibrosis and mucosal-based lymphoplasmacytic infiltration, which would reflect two-layer and three-layer structures of the gallbladder wall on the images, as described below. In addition, the report by Wang et al. [12] indicted that gallbladders in which the extramural inflammatory infiltrate outweighed the mucosal infiltrate were almost exclusively from patients with AIP, and not from those with primary sclerosing cholangitis and pancreatic cancer, which could also be clues for differential diagnosis.

## 3. Clinical Features of IgG4-Related Sclerosing Cholecystitis

According to our search with keywords “IgG4” or “immunoglobulin-4” and “cholecystitis”, or “gallbladder” on PubMed (1980 to May 2021), there have been 47 cases of IgG4-related sclerosing cholecystitis (IgG4-CC) with some information, including 9 cases of IgG4-CC with AIP (Table 1) and 11 cases of IgG4-CC without AIP (Table 2) that have sufficient clinical information [13,14,15,16,17,18,19,20,21,22,23,24,25,26,27,28,29,30]. The patient characteristics of the 47 cases are as follows: male/female ratio of 32/14 (unknown 1), mean age of 57.4, patients with/without autoimmune pancreatitis ratio of 15/11 (unknown 21), patients with/without gallbladder stone ratio of 11/28 (2 sludge and 6 unknown 6, and an average serum IgG4 level of 489.6 mg/dL in 14 cases (not assessed in 33 cases). According to the nationwide survey in Japan as well as a review article, the typical patient with IgG4-related disease is a middle-aged to elderly man [31,32]. For AIP, the mean age at diagnosis is 67 years and the male/female ratio is 3/1, which indicates that the average age of patients with IgG4-CC is younger and the proportion of female tends to be higher.

As past reports have shown that 25–32% of AIPs are associated with IgG4-CC [9], the nine cases of IgG4-CC with AIPs retrieved in this review are considered to be the tip of the iceberg. Namely, IgG4-CC can be more frequently associated with AIP than we expect it to be or than what has been published. IgG4-CC can also occur with sclerosing cholangitis and thickening of the gallbladder wall, which is detected upon imaging but is asymptomatic in most cases [8], as shown in Table 1 (six out of nine cases without IgG4-CC-related symptoms), and can lead to oversight and underestimates.

With regard to 11 rare cases of IgG4-CC without AIP, the patient characteristics are as follows: M/F of 9/2, average age of 58.9 (range of 18–76), and mean serum IgG4 level of 134 mg/dL (Table 2). According to a previous report, a higher serum IgG4 at diagnosis could be associated with multi-organ involvement and risk of relapse. [33] This would be why the serum IgG4 level is lower in the isolated IgG4-CC cases. In addition, unexpectedly, 9 out of 11 cases had some IgG4-CC-related symptoms (Table 2).

## 4. Image Features of IgG4-Related Sclerosing Cholecystitis

The fundamental finding in an image of IgG4-CC is the thickening of the gallbladder wall, which reflects fibrosis with a dense lymphoplasmacytic infiltrate. However, the finding of a thickened gallbladder wall is not specific for IgG4-CC, and must be differentiated from other diseases such as adenomyomatosis, cholecystitis caused by gallbladder stone/debris, xanthogranulomatous cholecystitis (XGC), and gallbladder cancer. In practice, special cases without AIP accompanied gallbladder adenomyomatosis in the IgG4-CC lesion that was confirmed by pathological examination have been reported [15,18,21].

In diagnostic imaging, we need to have some prior knowledge in order to differentiate between the diseases. First, we need to know whether the distribution of the location of IgG4-CC is specific or not. In the abovementioned 47 cases, the locations of the 41 IgG4-CC lesions were 4 in the neck of the gallbladder (Gn), 6 in the fundus of the gallbladder (Gf), 2 in the fundus to the body of the gallbladder (Gfb), and 29 diffuse. Namely, based on location, the lesions of IgG4-CC can be classified into the two types: the localized (Gn/Gf/Gfb) and diffuse type, which resemble the subtypes of gallbladder adenomyomatosis. About 70% of IgG4-CC diffusely developed in the whole gallbladder, while IgG4-CC at the neck of the gallbladder (Gn) developed in the cases without AIP alone (4 cases [24,25,27,30]), which all mimicked gallbladder cancer and were very difficult to be differentiated in the previous reports. Second, in the 47 cases, gallbladder stones/debris were not in the cases with AIP, which could be caused by the disturbance of bile flow by the Gn lesion or the misdiagnosis of the gallbladder wall thickening as the secondary change from stones/debris in some cases with AIP, as extrahepatic bile duct or pancreatic head lesions tend to be accompanied by debris. This is why there have been no reports of cases with both AIP and gallbladder stones. Gallbladder wall thickening with stones/debris should also be carefully observed for accurate diagnosis, in addition to the cases without stones/debris.

With this background information, we reviewed the image findings of IgG4-CC in previous reports including our cases [15,30], and would like to suggest clues for the image diagnosis of IgG4-CC, although this topic is never simple and can be complicated by some coexisting diseases such as choledocholithiasis and adenomyomatosis. Note that this review article is mainly based on available case reports, which could lead to the bias that this review includes more special cases than general cases.

### 4.1. Diffuse Type of IgG4-CC

**Figure 1:**
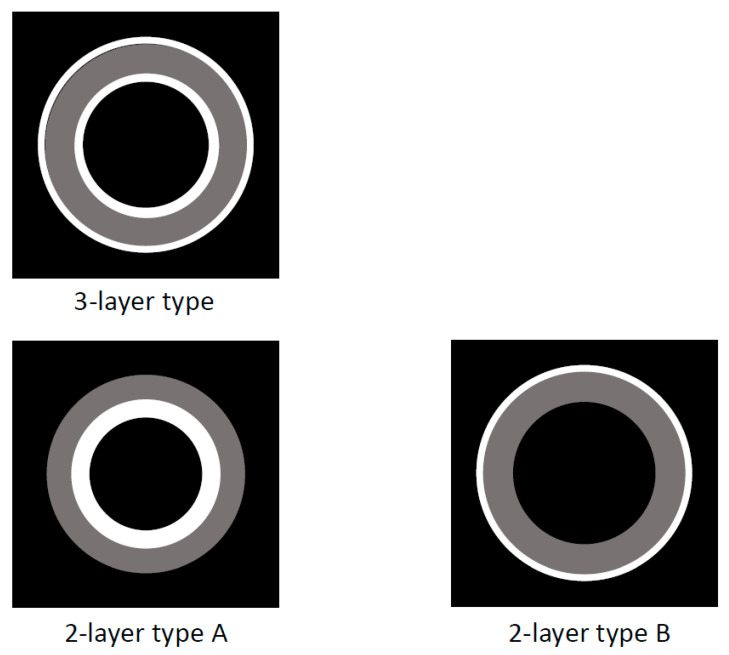
Schema of US/EUS findings in a diffuse type of IgG4-sclerosing cholecystitis. A diffuse type IgG4-CC could be classified into 3- or 2-layer type.

**Figure 2:**
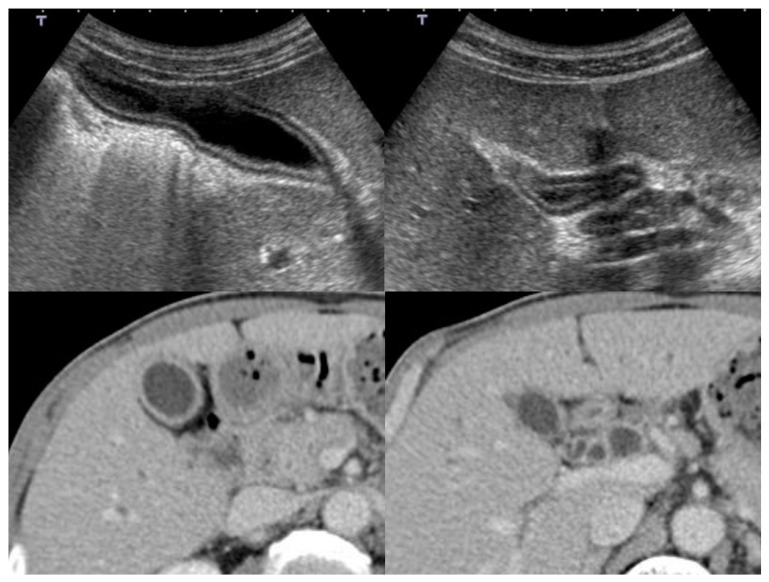
US (3-layer type) and CT (2-layer type) findings of a case with a diffuse type of IgG4-sclerosing cholecystitis.

**Figure 3:**
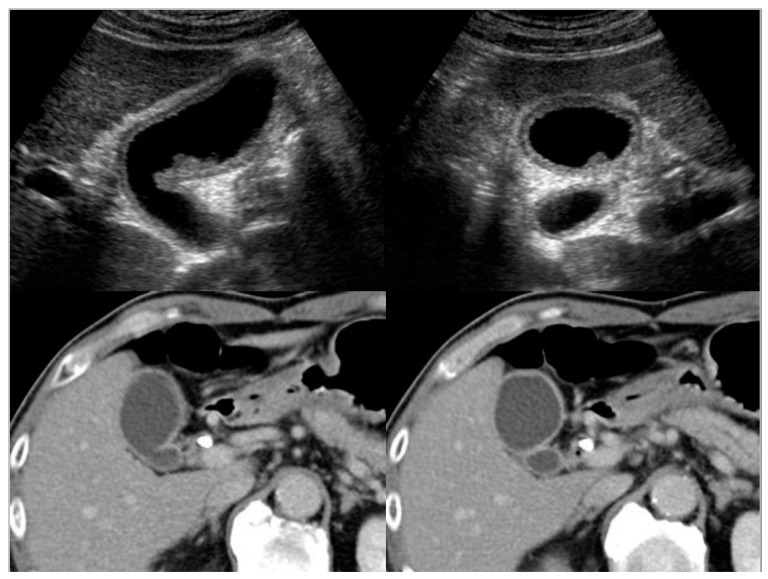
US (3-layer type) and CT (1-layer type) findings of a case with a diffuse type of IgG4-sclerosing cholecystitis.

**Figure 4:**
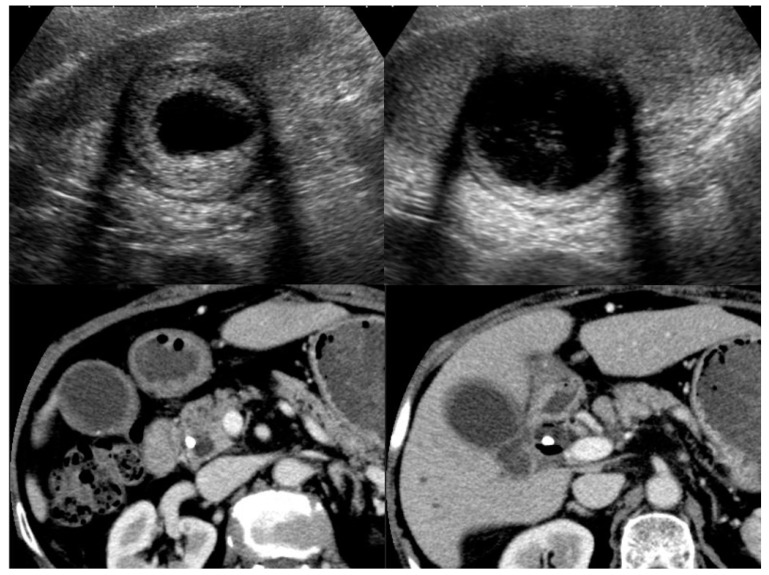
US (3-layer type) and CT (1-layer type) findings of a case with a diffuse type of IgG4-sclerosing cholecystitis.

Ultrasonography (US)/endoscopic ultrasonography (EUS) (Figure 1):

US/EUS is a standard imaging modality for the gallbladder because it is available everywhere, is noninvasive, and has a high-resolution for the depiction of the gallbladder. In reviewing previous reports on IgG4-CC, there have been some common image features (Figure 1). A diffuse type IgG4-CC could be classified into a three- or two-layer type, which reflects the gallbladder wall layer structure as well as the previous reports regarding the gallbladder wall, comparing US/EUS findings with histological specimens (hyper-hypo-hyper, hyper-hypoechoic/hypo-hyperechoic layers) [34,35]. In the three-layer type, the innermost layer (hyperechoic layer) includes the border between the gallbladder lumen and the mucosa (M), and the mucosa itself with lymphoplasmacytic infiltrate. The second layer (hypoechoic layer) includes the proper muscular layer (MP) and the fibrous layer of subserosa (SS), mainly exchanged by lymphoplasmacytic infiltrate with fibrosis. The outermost layer (hyperechoic layer) includes the adipose layer of “SS” with/without the lymphoplasmacytic infiltrate: this feature is found in the diffuse type IgG4-CC and also in the cases with AIP, whose typical pictures are shown in Figure 2, Figure 3 and Figure 4 and are described in the reports by Kamisawa et al. [11] and Matsubayashi et al [14]. In two-layer type A, as reported by Ishigami et al. [20], which is different from the US finding of the normal gallbladder wall [34] (two-layer type B), the inner layer (hyperechoic layer) includes “M” and “MP”, mainly exchanged by lymphoplasmacytic infiltrate with fibrosis, and the outer layer (hypoechoic layer) includes “MP”, and “SS” is mainly exchanged by lymphoplasmacytic infiltrate with fibrosis. In two-layer type B, as reported by Yamaguchi et al. [36] (Japanese article not searched on PubMed, but cited by Ichinokawa et al. [21]), the inner layer (hypoechoic layer) includes “M”, “MP”, and “SS”, mainly exchanged by lymphoplasmacytic infiltrate with fibrosis, and the outer layer (hyperechoic layer) includes “SS”.

**Figure 5:**
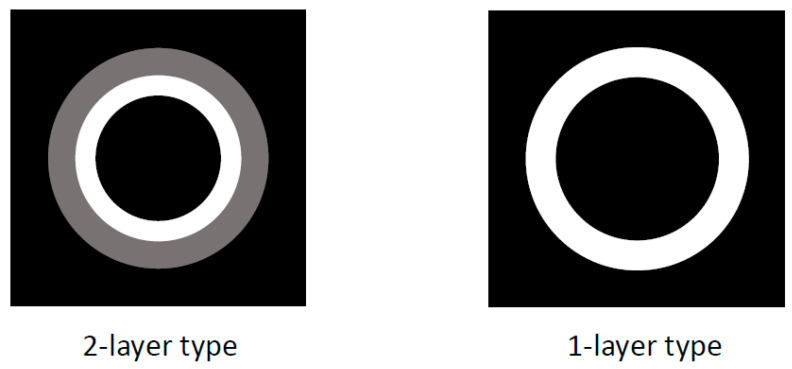
Schema of CT findings in a diffuse type of IgG4-sclerosing cholecystitis. A diffuse type IgG4-CC could be classified into 2- and 1-layer types.

Computed tomography (CT) (Figure 5):

CT is also a widely used imaging modality, although not as widely used as US because of its cost and radiation exposure issues, but it has the advantage of objectivity and high image resolution with vascularity information (Figure 5). Different from the US classification, a diffuse type IgG4-CC could be classified into two- and one-layer types, as well as common inflammatory gallbladder lesions such as acute cholecystitis and chronic cholecystitis with gallstones. The two-layer type is frequently observed in cases with an enlarged and significantly wall-thickened gallbladder. In the two-layer type, the almost normal mucosa with normal contrast enhancement (CE) or thickened mucosa with CE forms the inner layer with/without lymphoplasmacytic infiltrate and fibrosis; thickened “MP” and “SS” (and ‘S’) form the outer layer with almost no CE and lymphoplasmacytic infiltrate and fibrosis, whose typical pictures are shown in Figure 2 and are described in the reports by Leise et al. [22] and Jearth et al. [29]. In the one-layer type, “M”, “MP”, and “SS” (and “S”) form a smoothly thickened wall with CE reflecting lymphoplasmacytic infiltrate and fibrosis, whose typical pictures are shown in Figure 3 and Figure 4 and are described in the reports by Matsubayashi et al. [14] and Li et al. [17]. One rare case by Shin at al. showed a protruding B’ type, which was frequently observed in the localized type, as described below in detail.

Magnetic resonance imaging (MRI):

There have been only 3 reports [16,20,23] regarding MRI of IgG4-CC. In MRI, the wall of the gallbladder in IgG4-CC is thickened with lower intensity on T1-weighted images and slightly higher intensity on T2-weighted images compared with the liver parenchyma, and its luminal surface is smoothly depicted except for the IgG4-CC with gallstones.

### 4.2. Localized Type of IgG4-CC

**Figure 6:**
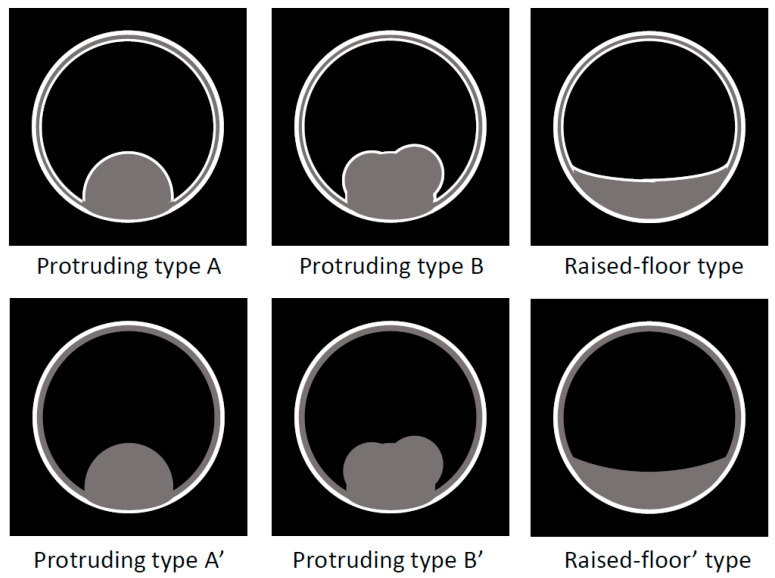
Schema of US/EUS findings in a localized type of IgG4-sclerosing cholecystitis. A localized IgG4-CC could be classed into the protruding type A (with smooth surface and innermost hyperechoic layer); -A’ (with smooth surface and no innermost hyperechoic layer); -B (with nodular surface and innermost hyperechoic layer); -B’ (with nodular surface and no innermost hyperechoic layer); raised-floor type (with innermost hyperechoic layer), raised-floor’ type (without innermost hyperechoic layer).

**Figure 7:**
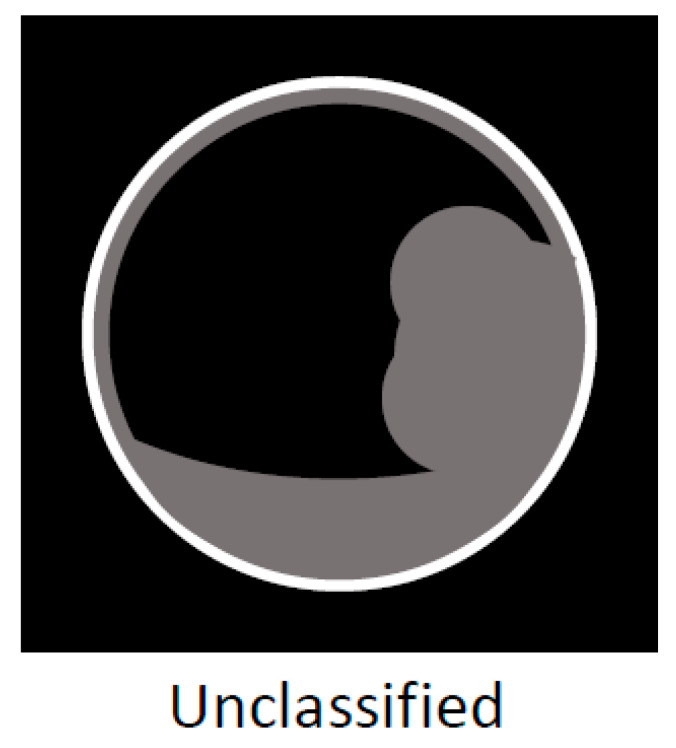
Schema of US/EUS findings in a localized type of IgG4-sclerosing cholecystitis: unclassified.

**Figure 8:**
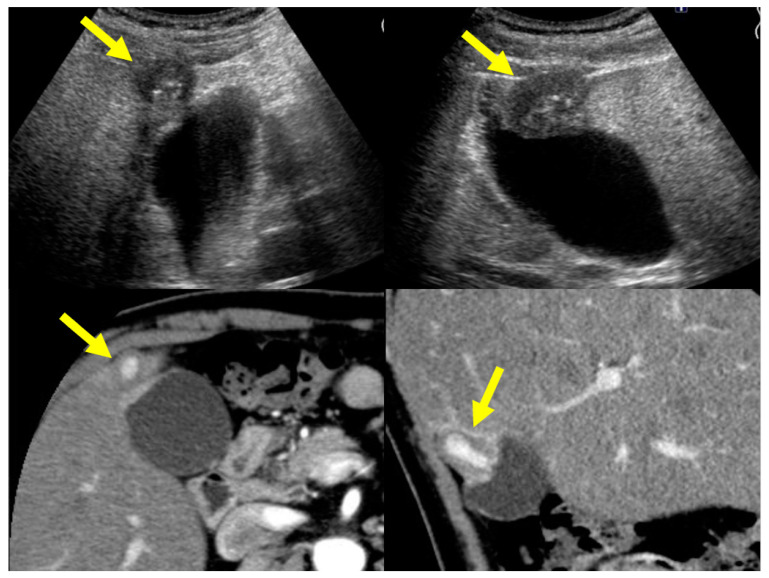
US (protruding type A) and CT (protruding type A) findings of a case with a localized type of IgG4-sclerosing cholecystitis (arrows).

**Figure 9:**
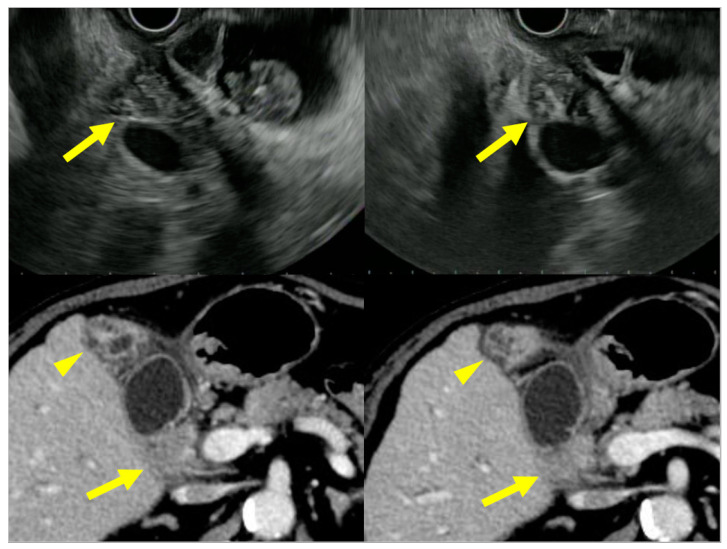
US (protruding type B’) and CT (protruding type B) findings of a case with a localized type of IgG4-sclerosing cholecystitis (arrows). The lesion at the fundus (arrowheads) was a true ADM lesion because this lesion had not changed at all after PSL treatment although a localized IgG4-CC lesion at the neck evidenced by biopsy had clearly improved.

**Figure 10:**
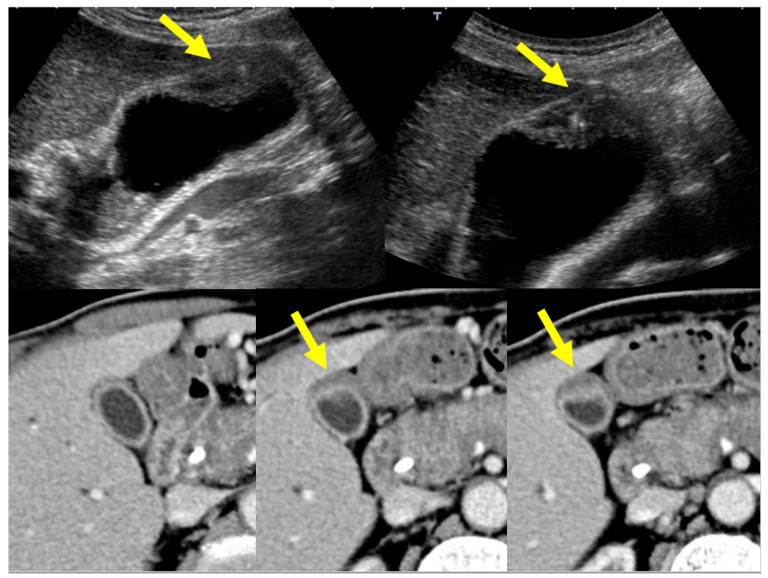
US (raised-floor type) and CT (raised-floor’ type) findings of a case with a localized type of IgG4-sclerosing cholecystitis (arrows).

US/EUS (Figure 6 and Figure 7):

Localized IgG4-CC can be classed into the following types: protruding type A (with smooth surface and innermost hyperechoic layer), which can protrude inwardly or outwardly, as shown in Figure 8 (our case reported by Kawakami et al. [15]); A’ (with a smooth surface and no innermost hyperechoic layer), as shown in the report by Inoue et al. [18]; B (with a nodular surface and innermost hyperechoic layer); B’ (with a nodular surface and no innermost hyperechoic layer), as shown in Figure 9 (our case reported by Nagai et al. [30]); raised-floor type (with innermost hyperechoic layer), as shown in Figure 10; raised-floor’ type (without innermost hyperechoic layer), as shown in the report by Ichinokawa et al. [21] and Gupta et al. [26] (Figure 6); and others (unclassified) (Figure 7). Each mass/nodule/thickened site harbors lymphoplasmacytic infiltrate and fibrosis, and occasionally reflects primary/secondary (which cannot be concluded at present) adenomyomatosis or xanthogranulomatous change. As a result, the image findings are very diverse and mimick gallbladder cancer. In five localized type cases (Table 1 and Table 2) with definite US/EUS findings, there were two cases with protruding type A/A’, one with protruding type B’, and two with raised-floor’ type.

**Figure 11:**
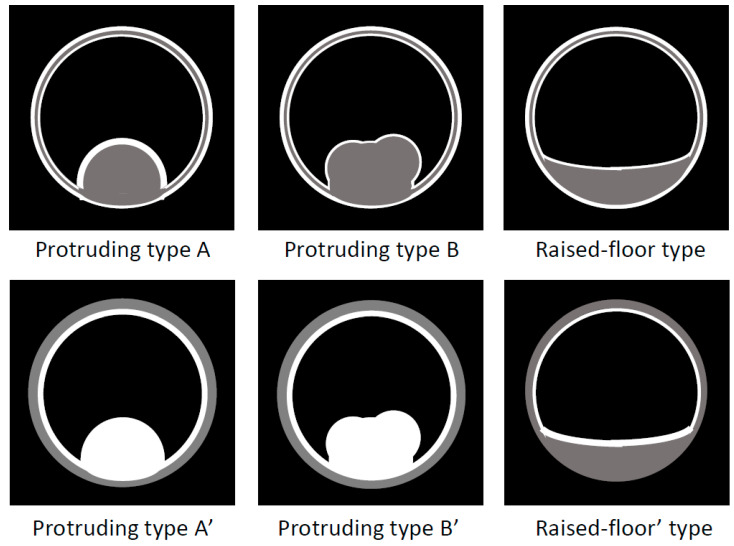
Schema of CT findings in a localized type of IgG4-sclerosing cholecystitis. A localized IgG4-CC could be classed into the protruding type A (with smooth and contrast-enhancing surface); -A’ (with smooth surface and homogenous CE); -B (with nodular and contrast-enhancing surface); -B’ (with nodular and contrast-enhancing surface lined by a homogenously thickened outer layer); raised-floor type (with contrast-enhancing surface); raised-floor’ type (without contrast-enhancing surface).

**Figure 12:**
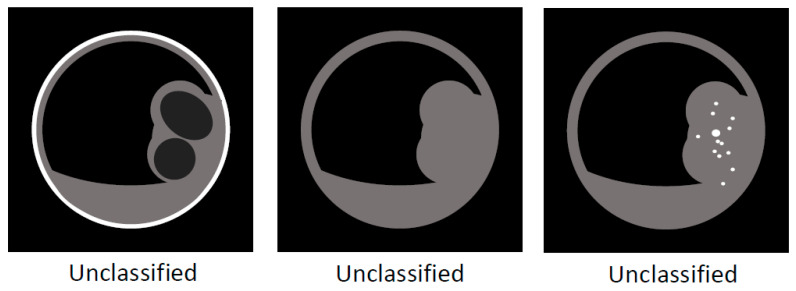
Schema of CT findings in a localized type of IgG4-sclerosing cholecystitis: others (not classified).

CT (Figure 11 and Figure 12):

Localized IgG4-CC can be classed into the following types: protruding type A (with smooth and contrast-enhancing surface), which can protrude inwardly or outwardly as shown in seven reports [13,15,24,25,27,28] including our case (Figure 8); A’ (with smooth surface and homogenous CE), as shown in the report by Inoue et al. [18]; B (with a nodular and contrast-enhancing surface), as shown in Figure 9 (our case reported by Nagai et al. [30]); B’ (with a nodular and contrast-enhancing surface lined by a homogenously thickened outer layer), as shown in the report by Shin et al. [23]; raised-floor type (with contrast-enhancing surface), as shown in the report by Ichinokawa et al. [21]; raised-floor’ type (without contrast-enhancing surface) as shown in Figure 10 (Figure 11); and others (diverse forms, scattered enhancement, thus unclassified) (Figure 12). In 11 localized type cases (Table 1 and Table 2) with definite CT findings, there were eight cases of protruding type A/A’, two of protruding type B/B’, one of raised-floor type, and one of other type.

Some of the IgG4-CC cases accompanied adenomyomatosis, xanthogranulomatous cholecystitis (XGC), or hyalinizing cholecystitis (HC), in addition to cholelithiasis, which can all modify and complicate the image findings of the gallbladder in ultrasonography and CT. Hong et al. [37] indicated that XGC shows overlapping histological features with IgG4-related cholecystitis, and that IgG4-CC (about 16%) could be diagnosed as XGC if careful inspection of the resected specimen of the gallbladder, in addition to cautious image diagnosis, was not performed. Gupta et al. [26] reported the possibility that some cases of HC represent the end stage of IgG4-related disease. However, as Ishigami et al. [20] also stated, the presence of wall thickening with the intact or smooth mucosal layer followed by a homogenously thickened outer (“MP” and “SS”) layer would be a helpful morphological finding to distinguish IgG4-CC from gallbladder cancer. As they indicated, US (or EUS) with contrast enhancement would be very effective to delineate the layer structure of the gallbladder wall, focusing on the smooth mucosal layer, especially if the gallbladder had stones or debris in the inner lumen.

## 5. Differential Diagnosis

Based on the image findings as described above, we could differentiate IgG4-CC from other gallbladder diseases to some extent. However, there were some diseases with similar image findings that occasionally made it difficult to differentiate IgG4-CC, as the previous reports indicated. We summarize the representative lesions to be differentiated from IgG4-CC as follows:

### 5.1. Gallbladder Cancer

Many cases with localized lesions and invasion to the hepatic parenchyma of IgG4-CC have been misdiagnosed as gallbladder cancer because most of the (98%) malignancy of the gallbladder is derived from the epithelium [38], and occasionally forms a polypoid lesion in the lumen or frequently invades into the hepatic parenchyma. Thus, IgG4-CC with an irregular mucosal layer, such as the case by Shin et al. [23], and polypoid gallbladder cancer with an irregular surface are extremely similar. In addition, gallbladder cancer can present a hypoechoic area in the deep layer with invasion, which resembles the thickened hypoechoic layer of the IgG4-CC lesion. In such a situation, a differentiating factor can be whether the smooth and regular mucosal layer exists or not.

### 5.2. Adenomyomatosis

IgG4-CC and adenomyomatosis can be similarly classified based on morphology (localized and diffuse type) and, more troublingly, IgG4-CC can be associated with adenomyomatosis and presents the same characteristic finding, namely dilatation of the Rokitansky−Aschoff sinus. However, the previous cases of IgG4-CC with adenomyomatosis are limited to the localized type of IgG4-CC [15,18,21], not to the diffuse type, which means that the diffuse type of adnomyomatosis can be excluded from the differential diagnosis of IgG4-CC at this stage.

### 5.3. Xanthogranulomatous Cholecystitis (Chronic Cholecystitis)

According to Hong et al. [37], XGC can overlap with IgG4-CC in histological features, and mass-forming lesions in XGC cases can accompany histological features suggestive of IgG4-related disease. It is known in autoimmune pancreatitis whether the terminal stage of AIP without treatment can present the same findings of chronic pancreatitis, such as the atrophy or calcification of the pancreatic parenchyma and pancreatic stone in the pancreatic duct. Thus, the results of Hong et al. [37] mean that IgG4-CC without treatment could progress to chronic cholecystitis, including XGC and AIP.

### 5.4. Malignant Lymphoma

Although malignant lymphoma of the gallbladder is extremely rare [39], image findings of malignant lymphoma and IgG4-CC can resemble and imitate each other because both of them originate from similar cell sources (lymphocytes and lymphoplasma cells) and mainly develop in the submucosal layer. Thus, the mucosal layer (innermost layer) is maintained (intact), and is smooth and lined by the thickened submucosal layer. However, the former consists of monoclonal cells and more high-density cell groups than the latter, while the latter (IgG4-CC) consists of polyclonal cells and more low-density cell groups than the former. Thus, the echogenicity of the malignant lymphoma lesion is extremely low [40] and would be lower than that of IgG4-CC.

### 5.5. Metastatic Neoplasm

Some neoplasms, including melanoma and carcinoma, can metastasize to the gallbladder and frequently form a polypoid lesion [41] covered by the normal gallbladder epithelium [42], which is occasionally observed in IgG4-CC. Many metastatic neoplasms take over the characteristics of original neoplasms and suggest similar vascularities on CT and other vascular imaging, which can be clues for a diagnosis [41]. For example, melanoma and renal cell carcinoma have a hypervascular feature and their metastatic lesions in the gallbladder have the same characteristics. Both hepatocellular carcinoma and metastasis to the gallbladder show early enhancement and early washout of contrast materials. For evaluation of vascularity of the lesions, CT has an advantage over US because of its objectivity.

## 6. Conclusions and Future Perspectives

For the diagnosis of IgG4-CC, US/EUS plays a key role, complemented by CT: the wall thickening with the intact or smooth inner layer followed by a homogenously thickened outer layer would be noteworthy and important findings.

In the near future, the diagnostic criteria of IgG4-CC should be established based on the abovementioned image features and pathological facts with the accumulation of IgG4-CC cases.

## Figures and Tables

**Table 1:** Previous reports on IgG4-related sclerosing cholecystitis with autoimmune pancreatitis.

Year	Author	M/F	Age	Symptom	Location	Serum IgG4 (mg/dl)	Infiltration to the Liver	Adjacent CBD Wall Thickening	GB Stone	US/EUS Type	CT Type
2005	Gumbs	F	68	Epigastralgia, fever, jaundice *, low appetite	Gf	NA	(-)	(-)	(-)	NA	Protruding A with 3-layer feature
2008	Matsubayashi	M	62	Jaundice *	Diffuse	764	(-)	(-)	(-)	3-layer	1-layer
2010	Kawakami §	M	55	None	Gf	455	(-)	(-)	(-)	Protruding A	Protruding A †
2013	Ishida	M	61	None	Diffuse	NA	(-)	(-)	(-)	NA	NA
2015	Li	M	61	Abdominal distension, pruritus *, jaundice *	Diffuse	1750	(-)	(+)	(-)	NA	1-layer
2015	Inoue §	F	60	None	Gf	813	(-)	(-)	(-)	Protruding A’	Protruding A’
2016	Yadav	M	68	Jaundice *, right-sided abdominal pain, weight loss	Diffuse	164	(-)	(-)	(-)	NA	NA
2018	Ishigami	M	82	None	Diffuse	943	(-)	(-)	(-)	2-layer A	NA
2019	Ichinokawa §	M	56	None	Gf	721	(-)	(-)	(-)	Raised-floor’	Raised-floor

M/F, male or female; CBD, common bile duct; GB, gallbladder; EUS, endoscopic ultrasonography; US, ultrasonography; NA, not assessed. * Associated with autoimmune pancreatitis. † Protruding type A with the high-density mucosal layer intruded into the nodule. § Adenomyomatosis was complicated in the fundus of gallbladder.

**Table 2:** Previous reports on IgG4-related sclerosing cholecystitis without autoimmune pancreatitis.

Year	Author	M/F	Age	Symptom	Location	Serum IgG4 (mg/dl)	Infiltration to the Liver	Adjacent CBD Wall Thickening	GB Stone	US/EUS Type	CT Type
2011	Leise	M	76	Right upper quadrant discomfort	Diffuse	134	(-)	(+)	(-)	NA	2-layer
2013	Shin	M	58	Right upper quadrant pain	Diffuse	NA	(+)	(-)	(+)	NA	Protruding B’
2013	Lee	M	59	Jaundice, right upper abdominal discomfort	Gn	75	(+)	(+)	(+)	NA	Protruding A †
2014	Feely ||	F	61	Jaundice, indigestion, acholic stools	Gn	17	(+)	(+)	(-)	NA	Others
2014	Feely ||	F	71	Right upper quadrant pain	Gf	(45)	(+)	(-)	(+)	NA	Protruding A ‡
2014	Feely	M	53	right upper quadrant and mid-epigastric pain	Gf	NA	(+)	(-)	(-)	NA	Protruding A ‡
2015	Gupta	M	76	None	Gf-Gb	436	(-)	(-)	(-) (debris)	Raised-floor’	NA
2015	Takahashi ||	M	18	jaundice, nausea, fatigue, loss of appetite	Gn	40	(-)	(-)	(-)	NA	Protruding A †
2019	Kulkarni	M	48	Upper abdominal pain	Gfb	After Sx: 63	(+)	(+)	(+)	NA	Protruding A
2020	Jearth	M	57	Intermittent right upper quadrant pain	Diffuse	261	(+)	(-)	(+)	NA	2-layer
2021	Nagai	M	70	None	Gn	282	(-)	(+)	(-) (debris)	Protruding B’	Protruding B

M/F, male or female; CBD, common bile duct; GB, gallbladder; EUS, endoscopic ultrasonography; US, ultrasonography; NA, not assessed; Sx, surgery. † Outwardly protruding type A with heterogeneous CE. ‡ Outwardly protruding type A with complex low-density area. || Xanthogranulomatous cholecystitis was complicated with IgG4-CC. Multifocal luminal abscesses and eroded foci associated with gall stone were also present.

## Data Availability

Data supporting reported results can be found in the references at the end of this article.
